# Endocytosis of Activated Muscarinic m2 Receptor (m2R) in Live Mouse Hippocampal Neurons Occurs via a Clathrin-Dependent Pathway

**DOI:** 10.3389/fncel.2018.00450

**Published:** 2018-11-30

**Authors:** Lisa Lambert, David Dubayle, Assia Fafouri, Etienne Herzog, Zsolt Csaba, Pascal Dournaud, Salah El Mestikawy, Véronique Bernard

**Affiliations:** ^1^Sorbonne Université, Université Pierre et Marie Curie UM 119 – CNRS UMR 8246 – INSERM U1130, Neurosciences Paris Seine – Institut de Biologie Paris Seine (NPS – IBPS), Paris, France; ^2^Université Paris Descartes – CNRS UMR 8119, Centre de Neurophysique, Physiologie et Pathologie, Paris, France; ^3^PROTECT, INSERM U1141, Université Paris Diderot, Sorbonne Paris Cité, Paris, France; ^4^Interdisciplinary Institute for Neuroscience, University Bordeaux, UMR 5297, Bordeaux, France; ^5^Interdisciplinary Institute for Neuroscience, CNRS, UMR 5297, Bordeaux, France; ^6^Department of Psychiatry, Douglas Hospital Research Center, McGill University, Montréal, QC, Canada

**Keywords:** internalization, G protein-coupled receptor, mouse, trafficking, time lapse confocal microscopy

## Abstract

Our aim was to examine the dynamics of the muscarinic m2 receptor (m2R), a G-protein coupled receptor (GPCR), after agonist activation in living hippocampal neurons, and especially clathrin dependency endocytosis. We have previously shown that the m2R undergoes agonist-induced internalization *in vivo*. However, the nature of the endocytotic pathway used by m2R after activation is still unknown in living neurons. Using live cell imaging and quantitative analyses, we have monitored the effect of stimulation on the fate of the membrane-bound m2R and on its redistribution in intraneuronal compartments. Shortly (6 min) after activation, m2R is internalized into clathrin immunopositive structures. Furthermore, after clathrin-dependent endocytosis, m2R associates with early and late endosomes and with subcellular organelles involved in degradation. Together, these results provide, for the first time, a description of m2R trafficking in living neurons and prove that m2R undergoes clathrin-dependent endocytosis before being degraded.

## Introduction

Most neurotransmitter and neurotransmitter-related drugs modulate neuronal activity through G-protein-coupled receptors (GPCRs). Mechanisms that control GPCR compartmentalization, including membrane availability, enable a neuron to adapt its response to local changes in neurotransmitter environment.

Ligand-induced endocytosis is characterized by the internalization of membrane molecules, including GPCRs, from the cell surface into internal membrane compartments. Endocytosis is a complex process that involves different steps. First, endocytosis of GPCRs, classically involves recruitment of agonist-occupied receptor into vesicles for entry into the endocytic pathway. This early vesicular trafficking can be divided into two main pathways: the classic, clathrin-mediated endocytic pathway and the atypical, clathrin-independent, that may be caveolin-1 or *flotillin*-1-enriched lipid-raft-dependent (Hansen and Nichols, 2009). Second, the cell may lead receptor containing vesicles to further endosomal processing through different subcellular compartments and may either recycle the GPCR back to the plasma membrane (PM) and/or degrade them. These early and late trafficking events mediate important functions for the neuron, tuning its responsiveness to ligands over both short-term and long-term periods and regulating receptor coupling to signal transduction pathways.

The molecular mechanisms underlying the endocytotic processing are still not clearly defined but are receptor-specific and may vary between cell types. For example, the highly related dopamine D1 or D2 receptors may have different internalization pathways (Vickery and von Zastrow, 1999). Intracellular signaling pathway may also be dependent of the cell type as shown for ErbB2 or 5-HT_1A_ receptor (Carrel et al., 2006; Hashizume et al., 2008). GPCR endocytosis studies have mostly been performed in cell lines and rarely in neurons. Yet, as polarized and arborized cells, neurons may display endocytosis features that serve their specific physiological functions, including receptor targeting to distinct subcellular compartments (McDonald et al., 2007a).

Our work focuses on the muscarinic receptor m2R, a metabotropic acetylcholine receptor involved in autoregulation of ACh release especially in the hippocampus and cortex (Zhang et al., 2002). In the present study, we have investigated the dynamics of the early endocytosis steps of the acetylcholine muscarinic m2 receptor (m2R) in live neurons. Indeed, the subcellular events after the stimulation of m2R may play a key role in the function of cholinergic neurons, especially in the regulation of their neuronal activity and/or in the inhibition of ACh release. We have previously shown that m2R displays endocytosis *in vivo* in striatal cholinergic neurons after acute stimulation (Bernard et al., 1998, 2006; Liste et al., 2002; Decossas et al., 2003). However, the precise endocytotic pathways used by m2R in living neurons are still unknown. One of the aims of our work was to determine whether m2R internalization occurs via a clathrin dependent pathway.

The m2R dynamics was investigated in hippocampal neurons after agonist activation using new fluorescent m2R fusion proteins N-terminally tagged with green fluorescent protein (GFP) or super-ecliptic pHluorin (SEP), a pH-sensitive chimera which facilitate the detection of surface receptor expression in live cells (McDonald et al., 2007b). Live-cell confocal imaging was used to visualize, analyze and quantify m2R dynamics. Real-time early trafficking events of the m2R were especially examined with regard to clathrin, a key protein of the endocytic pathway, and to other intraneuronal post-endocytic compartments.

## Materials and Methods

All relevant experimental procedures followed the guidelines of the European Communities Council Directive (86/809/EEC) regarding the care and use of animals for experimental procedures, and the Ministère de l’Agriculture et de la Forêt, Service Vétérinaire de la Santé et de la Protection Animale (permission no. A 94-028- 21), and were approved by the Regional Ethics Committee no. 3 of Ile-de-France region on Animal Experiments.

### DNA Constructs

Two plasmids, mM2-pcDPS and pRK-ssGFP-NK3 encoding the m2R and GFP-neurokinin 3 receptor, were used to generate the GFP-m2R construct. The m2R fragment was amplified from the mM2-pcDPS plasmid by PCR and introduced as a SalI/XbaI fragment in the pRK-ssGFP-NK3 plasmid to replace NK3 and to generate a pRK-ssGFP-m2R plasmid and obtain the N-terminal labeled version of the receptor. The GFP-m2R fragment was flanked upstream of GFP by an optimized artificial signal sequence derived from the human growth hormone [hGH1; a signal sequence (ss)] (McDonald et al., 2007b). This plasmid is designated as GFP-m2R throughout this paper. Another plasmid encoding for m2R tagged with the SEP, a pH dependent fluorochrome, was generated (SEP-m2R). The SEP fragment was amplified from the SEP-TOPO plasmid by PCR and introduced as a BGlII/SalI fragment in the GFP-m2R plasmid to replace GFP and to generate a pRK-ssSEP-m2R plasmid. This plasmid is designated as SEP-m2R throughout this paper. The mM2-pcDPS, pRK-ssGFP-NK3, and SEP-TOPO were generous gifts from J. Wess (NIH, Bethesda, United States), A. Irving (University of Dundee, United Kingdom) and J. Henley (University of Bristol, United Kingdom), respectively. Alternatively, we have removed GFP from the pRK-ssGFP-m2R plasmid to produce a pRK-ss-m2R plasmid that coded for the wild-type m2R that was used to check the absence of negative effects of GFP in the endocytotic processes. This plasmid is designated as WT-m2R throughout this paper. The integrity of the constructs was confirmed by sequencing. CAV1-mCherry was a gift from Ari Helenius (Addgene plasmid # 27705, McDonald et al., 2007b).

### Neuronal Cultures and Transfections

Post-natal day 0 C57BL/6 mice were euthanized by decapitation. Hippocampi were dissected from mouse brains and dissociated in *Hanks’ Balanced Salt Solution* (HBSS) with papaine (Worthington Biochemical Corp. Lakewood, NJ, United States; 9001-73-4; 25 U/ml). Hippocampal neurons were plated on glass coverslips previously coated with Poly-L-Lysine 0.01% (Sigma-Aldrich, St. Louis, MO, United States). Neurons were grown in Neurobasal A medium supplemented with 2% B27, 1% glutamax, and 0.5% penicillin-streptomycin (Life Technologies; 10888022; 35050038; 17504044; 15140122, respectively), and maintained in an incubator with 5% CO_2_. Hippocampal neurons were transfected at day *in vitro* (DIV) 7 with the appropriate cDNA (WT-m2R, GFP-m2R, and SEP-m2R) using Lipofectamine 2000 (Life Technologies; 11668019) in OptiMEM medium (Life Technologies; 31985-062). All experiments were performed the day after transfection (DIV8). The m2R is not constitutively expressed by hippocampal neurons in culture.

### Pharmacological Treatments

The effect of a muscarinic receptor agonist carbamylcholine, further referred to as “carbachol” (CCh) (Sigma-Aldrich, St. Louis, MO, United States), on m2R trafficking was observed in hippocampal neurons.

For real-time experiments, the imaging chamber was perfused 1–120 min with 30 or 100 μM CCh diluted in the isotonic medium. In some experiments, neurons were perfused with 10 nM of the muscarinic receptor antagonist atropine (Sigma-Aldrich, St. Louis, MO, United States), 10 min prior to 100 μM CCh. CCh was added then together with atropine. In order to reveal receptors associated with acidic intraneuronal organelles after SEP-m2R transfection, NH4Cl (50 mM) was added in the perfusion bath.

For other experiments, neurons were incubated with 30 or 100 μM CCh in Neurobasal medium for 3, 6, 20 min, 1 or 2 h and fixed with 2% paraformaldehyde for 5 min. In some experiments, neurons were perfused with 10 nM of the muscarinic receptor antagonist atropine (Sigma-Aldrich, St. Louis, MO, United States) 10 min prior to CCh and then during endocytosis 15 min together with 30 μM CCh.

### Clathrin-Dependent Endocytosis Blockade by Molecular Manipulation of a Selected Clathrin-Dependent Endocytosis Pathway Protein

To block the clathrin-dependent route in the endocytic pathway, we blocked the function of a key protein in the endocytic pathway, Eps15, by expressing dominant-negative proteins, fused to GFP. DIII and EH29 mutants were generated by deleting distinct parts of the DNA coding for Eps15 (Benmerah et al., 1999). Plasmid constructs of dominant negative Eps15 (DIII and EH29) and control (D3Δ2) were kindly provided by A. Benmerah (Paris University, Imagine Institute, Paris, France). Neurons were transiently co-transfected with dominant negative plasmids and the WT-m2R plasmid using Lipofectamine 2000 (Life Technologies, Saint Aubin, France). Neurons expressing simultaneously Eps15 mutants or control (identified by GFP staining) and m2R (identified by m2R ICC) were analyzed.

### Al594-Tf Uptake Used as a Marker of Clathrin-Mediated Endocytosis in Hippocampal Neurons

Transferrin uptake occurs through a clathrin-mediated endocytosis (CME) (Benmerah et al., 1999).

In order to know wether m2R is internalized with the same pathway, we have studied the colocalization of fluorescence for m2R and Al594-Tf in untreated neurons and after CCh stimulation. For that, the neurons were incubated with Al594-Tf alone for 10 min, then with or without CCh for 15 min. After fixation, neurons were observed under the confocal microscope and the colocalization of fluorescent m2R ICC signal and Al594-Tf was analyzed using the Jacop ImageJ Plugin (see below).

### Antibodies and Immunocytochemistry

#### Antibodies

The m2R expressed after transfection with the WT-m2R plasmid was immunolocalized using a monoclonal anti-m2R antibody raised in rat against an intracellular epitope of the receptor (rat, Chemicon, Cat# MAB367, Lot# **RRID**: AB_94952). The antibody recognized a single band on Western blots corresponding to the m2i3-GST fusion protein (Levey et al., 1995). In immunohistochemistry, it exhibited a pattern identical to that seen previously with polyclonal antibodies against the same antigen (Levey et al., 1991, 1995). No immunoreactivity was seen when the antibody was used on tissue from m2R knockout mice (Duttaroy et al., 2002).

In some experiments, GFP or SEP expressed after transfection with the GFP-m2R or SEP-m2R plasmids was detected using a anti-GFP antibody (mouse, Roche Applied Science Cat# 11814460001, Lot# **RRID**:AB_390913). To identify subcellular organelles associated with m2R after stimulation with CCh for 3, 6, or 20 min, 1 or 2 h, the following antisera were used: anti-Clathrin heavy chain (CHC; mouse; BD Biosciences Cat# 610499 Lot# **RRID**:AB_397865); anti-Golgi matrix protein of 130kDa (GM130; mouse; BD Biosciences Cat# 610822 Lot# **RRID**:AB_398141); anti-Rab5 (mouse; BD Biosciences Cat# 610281 Lot# **RRID**:AB_397676); anti-Rab9 (mouse; Thermo Fisher Scientific Cat# MA3-067 Lot# **RRID**:AB_2175599); anti-protein disulphide isomerase (PDI; mouse; Thermo Fisher Scientific Cat# MA3-019 Lot# **RRID**:AB_2163120); anti-cathepsin D (CathD (G-19); mouse; Santa Cruz Biotechnology Cat# sc-6494 Lot# **RRID**:AB_2087097). Secondary antibodies used were donkey anti-rat Alexa568-conjugated or goat anti-rat Alexa488- (m2R, Thermo Fisher Scientific Cat# A-11077, **RRID**:AB_2534121 or Molecular Probes Cat# A-11006, **RRID**:AB_141373, respectively), and goat anti-mouse Alexa488-conjugated (GFP, (Thermo Fisher Scientific Cat# A32723, **RRID**:AB_2633275) or goat anti-mouse Alexa688-conjugated (GFP, CHC, GM130, Rab5, Rab9, PDI, Molecular Probes Cat# A-11004, **RRID**:AB_141371).

#### Immunocytochemistry

Neurons were fixed with 2% paraformaldehyde for 5 min at room temperature. The cells were washed in PBS and incubated 30 min with 4% normal donkey serum (Sigma-Aldrich, St. Louis, MO, United States). Primary antibodies were diluted in PBS with 1% normal donkey serum and 0.075% saponin and incubated overnight at 4°C. Neurons were washed in PBS and subsequently incubated with fluorescence-coupled secondary antibodies diluted in PBS with 0.075% saponin for 1 h at room temperature. Finally, cells were washed in PBS and mounted in Prolong gold (Thermo Fisher Scientific).

### Time-Lapse Imaging of Cultured Hippocampal Neurons

Time-lapse imaging was used to analyze (1) the pH-dependence of the SEP-m2R construct, and (2) the effect of CCh on the membrane associated m2R.

Time-lapse sequences from cultured hippocampal neurons transfected with selected plasmids were collected using a Leica DMI6000B inverted microscope (Leica Microsystems, Deerfield, IL, United States) equiped with a Yokogawa CSU-X1 spinning disc confocal head (Roper Scientific, Lisses, France) and a 100 mW 491 and 561 nm laser controlled by MetaMorph (Molecular Devices, St. Grégoire, France). The setup was enclosed in a thermal incubator set to 37°C under 5% CO_2_. Images were collected through a 63 × /1.4 numerical aperture oil-immersion objective and an additional 2 × lens on a QuantEM:512SC EMCCD (Photometrics, Tucson, AZ, United States).

For validation of SEP pH-dependence, hippocampal neurons transfected with the SEP-m2R were observed under the spinning disk microscope for 30 min and the pH of the medium was changed [7.4 to acidic pH (around 6.0)] time to time with or without NH4Cl (50 mM). Stacks of images were collected every 30 s for 30 min. In order to analyze the effect of CCh on the membrane associated m2R, hippocampal neurons transfected with the SEP-m2R were observed under the spinning disk microscope for 120 min and stacks of images were collected. Stacks of images were acquired every 30 s for 30 min. Images were treated using Fiji (Schindelin et al., 2012) and Adobe Photoshop softwares.

### Imaging of Fixed Cultures by Confocal Microscopy

Images were acquired on a Leica SP5 confocal system (Leica Microsystems, Deerfield, IL, United States). *z*-Series stacks of confocal images were acquired at 1024 × 1024 pixel resolution, with a pinhole setting of one Airy unit and optimal settings for gain and offset. For double immunolabeling quantifications, images were taken with a 63 × /1.4 numerical aperture (N.A.) Plan-Apochromat, an argon laser at an excitation wavelength of 488 nm, and a diode 561 nm or two diodes at 561 and 633 nm. Images were treated using Fiji (Schindelin et al., 2012) and Adobe Photoshop softwares.

### Quantification and Statistical Analyses

#### Quantification of Colocalization of Fluorescence

The quantification of colocalization of m2R with GFP or SEP (for validation of the constructs) and m2R with clathrin or organelle markers was analyzed with the “Just Another Colocalization Program” (JACoP) Plugin (ImageJ, National Institutes of Health), and statistical data are reported from the Costes’s randomization-based colocalization module (Bolte and Cordelières, 2006). Costes’s randomization method for measurement of colocalization was used to confirm, with >95% certainty, that the colocalization observed between the m2R and clathrin or organelle immunofluorescent signals was not caused by chance coincidence (Costes et al., 2004). A Pearson’s coefficient (pc) was calculated. Costes**’s** randomization was applied on five neurons from four mice of each genotype using at least 150 iterations per image. For validation of the constructs, analyses were performed on somatic areas and on the neuropile. For the colocalization of m2R with clathrin and organelles markers, analyses have been restricted to the somatic area. Just individual images (and not stacks of images) were analyzed. The quantification of colocalization was performed from the labeling on images observed with the 63× objective (surface of the field: 655 μm^2^).

The *pc* calculated in colocalizations analyses in WT and stimulated neurons were compared using a Mann–Whitney *U*-test or the Kruskal–Wallis test followed by Dunn’s Multiple Comparison Test when more than two groups had to be compared. All data are shown as the means ± SEM; NS, not significant; ^∗∗∗^*p* < 0.0001.

#### Quantification of the Density of m2R Clusters in Mutants of Eps 15

Hippocampal neurons were observed using the 63x objective and acquisitions were performed under the confocal microscope. Intracellular immunofluorescent clusters, representing m2R in endosomes, were segmented and counted using the FIJI/ImageJ software. Results are expressed as intracellular immunofluorescent clusters per μm^2^ cytoplasmic surface in Eps15 dominant negative-treated and control neurons.

#### Quantification of variation of fluorescence in time lapse experiments

The quantification of variation of fluorescence levels with time was automatically performed using the Fiji software. Mean intensity measurements following background subtraction from the whole neuron were pooled for each cell. Control values corresponded to the mean fluorescence intensity immediately at the beginning of the experiment or before addition of the muscarinic agonist. Data were compared using the repeated ANOVA test followed by the Dunnet *post hoc* test comparing each value to the value at the beginning of CCh treatment. The *p*-values are ^∗^*p* < 0.05; ^∗∗^*p* < 0.001; ^∗∗∗^*p* < 0.0001. The quantification of the number of clusters of internalized m2R per surface of neuron after CCh treatment was performed on projections of the stacks images. Data were compared using the repeated ANOVA test followed by the Dunnet *post hoc* test comparing each value to the value at 0 min or the Mann–Whitney *U*-test when the data were unpaired. The *p*-values are ^∗^*p* < 0.05; ^∗∗^*p* < 0.001; ^∗∗∗^*p* < 0.0001.

All the experiments have been replicated at least three times. For quantitative studies, 15–25 neurons per group were analyzed.

## Results

### Validation of the GFP-, SEP-, and WT-m2R Constructs

#### Expression of GFP-m2R and SEP-m2R in Living and Fixed Neurons

**FIGURE 1:**
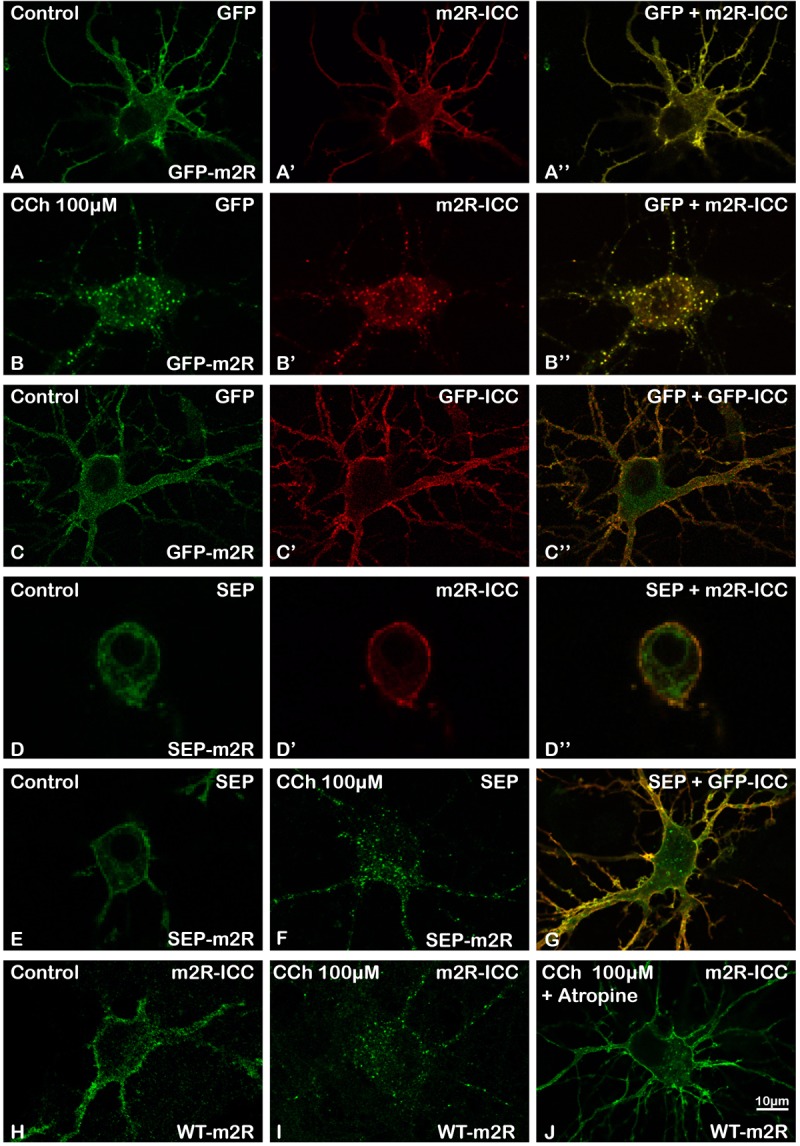
Validation of GFP-m2R, SEP-m2R, and WT-m2R expressing vectors and localization of transfected m2R in hippocampal neurons. Hippocampal neurons were transfected with a plasmid encoding the GFP-tagged receptor (GFP-m2R) **(A–C”)**, SEP-tagged receptor (SEP-m2R) **(D–G)** or wild-type receptor (WT-m2R: **H–J**), fixed, and processed for visualization of the receptor by confocal microscopy. Equatorial images of neurons (0.5 μM in depth) were selected and illustrated on this panel. The m2R localization was identified by GFP **(A,A”,B,B”,C,C”)** or SEP **(D,D”E,F,G)** native fluorescence or by fluorescent ICC using an anti-m2R (m2R-ICC : **A’,B’,D’,H–J**) or anti-GFP antibody (GFP-ICC : **C’,G**). Whatever the construction, the fluorescent signal is localized at the membrane of the soma and the dendrites. A faint signal is detected in the cytoplasm. **(B,F,I)** The stimulation with a muscarinic receptor agonist [Carbachol (CCh), 100 μM] induces a huge decrease of the signal at the somatic and dendritic membranes and a the appearance of a punctiform labeling in the cytoplasm when using a GFP **(B)** or SEP-tagged **(F)** or WT **(I)** construction. **(J)** A neuron, that has been pre-incubated with Atropine (10 nM), a muscarinic receptor antagonist, display a membrane labeling at the soma and dendrites similar to a control staining. **(A–B”,D–D”)** Fluorescent signals detected by direct visualization of GFP **(A,B)** or SEP **(D),** and by m2R ICC **(A’,B’,D’)** in a same neuron perfectly colocalize **(A”,B”,D”)**. **(C–C”)** GFP or SEP detection by ICC **(C’,G)** display a membrane labeling in a non-permeabilized neuron that colocalizes with the direct GFP or SEP fluorescence **(C,C”,G)**.

To analyze the dynamics of GPCRs with high resolution in fixed and living neurons, the m2R was tagged at the N terminus with GFP or SEP. By live or fixed-cell confocal microscopy, we have detected a predominant PM distribution of GFP-m2R or SEP-m2R fluorescence (Figures 1A,A”,C,C”,D,D”,E,G) in the cell body and the proximal and distal dendrites of hippocampal neurons. This distribution was similar to that detected for the m2R using a third intracellular loop directed antibody (Figures 1A’,A”,D’,D”) or to the endogenous receptor in hippocampal neurons (Bernard et al., 2003). Cytoplasmic fluorescence signal was low. Similar labelings were observed after transfection with the WT-m2R plasmid and immunocytochemistry with an m2R antibody (Figure 1H). The analysis of the colocalization of GFP with m2R-ICC and SEP with m2R-ICC was performed on fixed neurons using the Jacop Plugin of ImageJ are reported from the Costes’s randomization-based colocalization module (see Materials and Methods). The high Pearson’s coefficient at or higher than 0.8 (0.8707 ± 0.0171, *n* = 22; 0.8489 ± 0.0199, *n* = 25, respectively) confirmed the validation of the GFP-, SEP-, and WT-m2R constructs.

#### GFP and SEP Are Correctly Addressed to the External Plasma Membrane

In order to check that the m2R was correctly folded and addressed as expected to the PM with its N terminus at its extracellular side, we have co-detected GFP and SEP by their native fluorescence and by immunocytochemistry using an anti-GFP antibody. GFP immunofluorescence on fixed and unpermeabilized neurons, was restricted to the PM in perikarya and dendrites (Figures 1C’,C”). The analysis of the colocalization of GFP with GFP-ICC and SEP with GFP-ICC using the Jacop Plugin of ImageJ gave a high Pearson’s coefficient (0.8006 ± 0.0266, *n* = 14 and 0.7696 ± 0.0612, *n* = 7, respectively) confirmed the validation of the GFP-, SEP-, and WT-m2R constructs.

#### Surface Expression of GFP-m2R and SEP-m2R Is Dynamically Regulated by Agonist Exposure

As for other GPCRs, the m2R is internalized from the cell surface following agonist binding (Bernard et al., 1997, 1998, 2006). We especially checked that GFP or SEP did not disturb m2R internalization. The GFP-m2R and SEP-m2R underwent a time-dependent loss of cell surface receptors following CCh (100 μM) exposure in agreement with data for endocytosis of wild-type m2R expressed in neurons (Figures 1B,B’,B”,F; Bernard et al., 1998, 2003, 2006). Internalization was also observed when neurons were transfected with the WT-m2R plasmid and the m2R detected by immunocytochemistry with an m2R antibody (Figure 1I).

#### Validation of pH-Dependence of SEP-m2R

**FIGURE 2:**
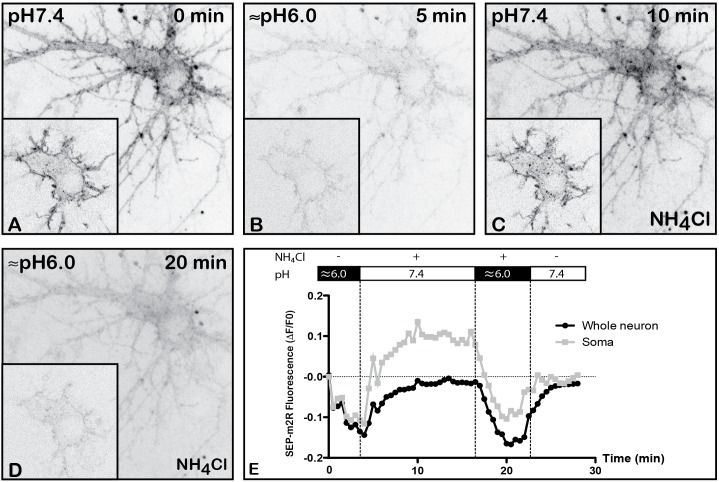
Validation of pH-dependence of SEP-m2R. A living hippocampal neuron transfected with SEP-m2R was observed by spinning disk confocal microscopy for 30 min. A stack of 20 images (0.5 μM in depth) were collected at 30 s intervals. A projection of the stack images was performed and an equatorial image was extracted (insert) and illustrated on this panel **(A–D)**. The effect of pH was observed on the fluorescence level with or without NH4Cl. **(A)** At pH7.4, the SEP-m2R is detected at the membrane of cell body and proximal dendrites. **(B)** At acidic pH, SEP-m2R labeling strongly decreases. **(C)** The SEP-m2R labeling is seen again the plasma membrane when the medium is back to pH7.4. When NH4Cl (50 mM) that is known to reveal receptors associated with acidic intraneuronal organelles is added in the medium, punctiform m2R labeling was also seen in the cytoplasm. **(D)** At acidic pH with NH4Cl, the SEP-m2R labeling is very weak again. **(E)** Quantification of the fluorescence level. Fluorescence was measured at the level of the whole neuron (corresponding mainly to plasma membranes) and in soma using the Fiji software. Data are expressed as normalized values compared to the fluorescence level at pH7.4 at 0 min. SEP-m2R labeling strongly decreases at acidic pH at plasma membranes and in the cytoplasm. NH4Cl at pH7.4 induces an increase of the staining close to the control values at the plasma membranes and much higher in soma. The acidic pH with NH4Cl strongly decreases fluorescence at membranes and in soma. The recovery of SEP-m2R fluorescence is shown in both compartments when the medium is back to pH7.4.

**FIGURE 3:**
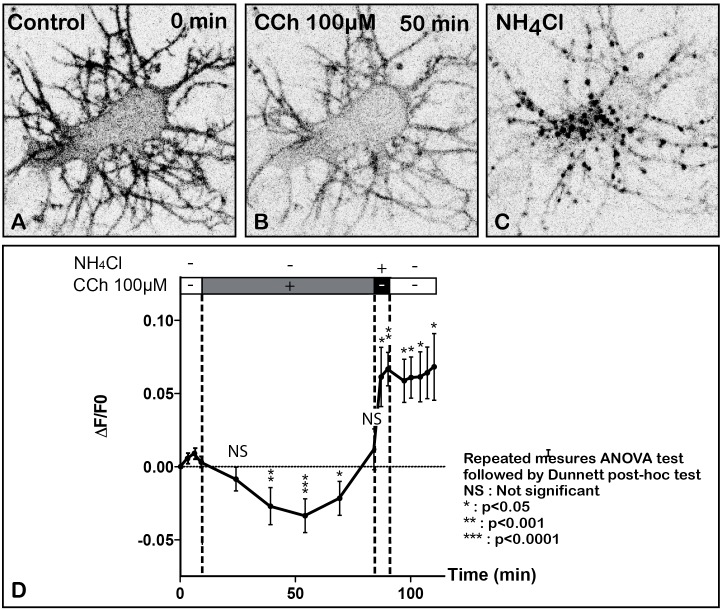
Time lapse imaging and quantification of SEP-m2R membrane labeling in a living neuron after stimulation by CCh, a muscarinic receptor agonist. A living hippocampal neuron transfected with SEP-m2R was observed by spinning disk confocal microscopy for 75 min. A stack of 20 images were collected at 30 s intervals. An equatorial image (0.5 μM in depth) was selected and illustrated on this panel **(A–C)**. **(A)** In control condition, the SEP-m2R staining is detected at the membrane of the cell body and proximal dendrites. A faint signal is also shown in the cytoplasm. **(B)** CCh (100 μM) induces a decrease of SEP-m2R labeling at cell body and dendrites levels. **(C)** Application of NH4Cl (50 mM) that reveals receptors associated with acidic intraneuronal organelles induces an abundant and intense punctiform staining in the cytoplasm. **(D)** Quantification of the effect of CCh on the fluorescence level +/- SEM using the Fiji software. Fluorescence was measured on three different neurons on a projection of the stack images at the level of the whole neuron using the Fiji software. Data are expressed as normalized values compared to the fluorescence level at 9 min before CCh application. The quantification shows a significant difference of m2R fluorescence with time. The statistical analysis (Repeated measures ANOVA test followed by the Dunnett *post hoc* test), performed on raw data, shows that CCh induces a significative decrease of fluorescence 39, 54, and 69 min after the beginning of the treatment. *Post hoc* anlayses were performed on two segments of the slop to analyze (1) the effect of CCh (from *T* = 0 min until 84 min) and (2) the effect of NH4Cl (from *T* = 84 min until 120 min) on fluorescence levels. The values are compared to the values at *T* = 9 min, the initiation point of CCh application for the CCh effect and at *T* = 84 min, the initiation of NH4Cl application, for NH4Cl effect. Results show a significant decrease of the fluorescent level from 39 to 54 min after CCh stimulation. From 54 min, fluorescence slowly returns to normal values. In contrast, NH4Cl, which reveals m2R attached to acidic vesicles, induces a significant increase of SEP-m2R fluorescence levels. NS, not significant, ^∗^*p* < 0.05; ^∗∗^*p* < 0.001; ^∗∗∗^*p* < 0.0001.

Although GFP is useful to report receptor localization, it is not possible to distinguish between surface and intracellular receptors in live cells using fusion proteins incorporating GFP. However, SEP, a pH-sensitive variant of GFP can be used to report surface expression, when expressed at an extracellular site (Miesenbock et al., 1998; Ashby et al., 2006; McDonald et al., 2007b). Genetically encoding SEP into the extracellular domain of a membrane protein of interest positions the fluorophore on the luminal side of the endoplasmic reticulum (ER) and in the extracellular region of the cell. SEP is fluorescent when the pH is greater than 6, but remains in an off state at lower pH values. Therefore, receptors tagged with SEP fluoresce when residing in the ER or upon insertion in the PM but not when confined to a trafficking vesicle. We therefore generated a SEP-m2R chimera by switching GFP for SEP in the original GFP-m2R construct. The pH dependence of SEP-m2R fluorescence was characterized in transfected hippocampal neurons. At physiological pH 7.4, SEP exhibits similar fluorescence to wild-type GFP (Figures 1A, 2A, 3A). A strong membrane-associated fluorescence was observed in the whole dendritic arborization and in the cell body. A faint staining was seen in the cytoplasm. When the pH is decreased to 6, the dendritic and cell body membrane labeling strongly decreases as well as the intracytoplasmic staining (-81 and -98%, respectively) (Figures 2B,E). Changes in fluorescence levels are reversible when the pH is back to 7.4 (Figures 2C,E). Also, SEP-m2R fluorescence is visible again at the PM of dendrites and cell body (Figure 2C). The quantification shows that SEP-m2R fluorescence is 10% under control (pH 7.4) values in the whole neuron (Figure 2E). Administration of NH4Cl (50 mM), a compound that equilibrates luminal pH of acidic intracellular vesicles to the extracellular neutral pH, reveals labeling in some intracytoplasmic organelles when the pH is back around 6.0–7.4, and the SEP-m2R fluorescence is 59% above control values (Figures 2C,E). In contrast, when the extracellular medium, including intravesicular pH (due to NH4Cl presence) is set to a more acidic pH (around 6.0), SEP-m2R labeling disappears at membranes and in the soma [-87 and -84% (Figures 2D,E)].

#### Specificity of Activation of Muscarinic Receptors by Carbachol

Receptor internalization induced by CCh was totally blocked in the presence of the muscarinic receptor antagonist atropine (10nM) (Figure 1J).

All validation experiments were performed on three independent cultures, and in each culture, at least 10 neurons were analyzed.

### Dynamics of m2R Internalization

**FIGURE 4:**
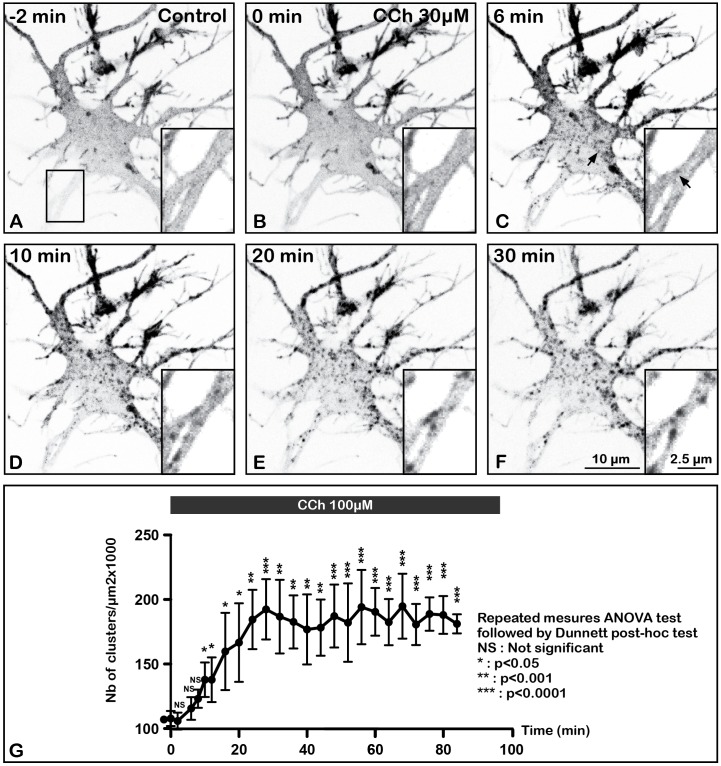
Time lapse imaging and quantification of internalization of m2R in a living neuron after stimulation by CCh, a muscarinic receptor agonist. A living hippocampal neuron transfected with GFP-m2R was observed by spinning disk confocal microscopy before and during a 30 min long carbachol (100 μM) treatment. A stack of 20 consecutive confocal images (0.5 μM in depth) were acquired every 30 s. A projection of the stack images was performed and illustrated on this panel **(A–F)**. An equatorial image was selected and an enlargement of a dendritic shaft is shown at the bottom of each image (insert). Here is shown the GFP-m2R labeling in this neuron 3 min before CCh and every 3 min for 30 min. Before CCh addition **(A)** and at the beginning of agonist treatment **(C)**, m2R was detected mainly at the plasma membrane of soma and dendrites. Agonist induces internalization of membrane-associated m2R and clusterization 6 min after treatment initiation in the cytoplasm of soma and dendrites (arrows in **C**). **(G)** Quantification of the effect of CCh on the density of fluorescent clusters +/- SEM in the cytoplasm of 3 neurons. Fluorescence was measured on a projection of the stack images using the Fiji software. The clusters density increases during the first 30 min and stabilized afterward. The statistical analysis (repeated measures ANOVA test followed by the Dunnett *post hoc* test) shows a significant increase of the number of clusters as early as 10 min after the beginning of CCh stimulation.

To investigate the dynamic properties of the m2R after agonist activation, we have developed a combination of experiments using individual live hippocampal neurons transfected with the GFP-m2R or SEP-m2R plasmids and exposed to 100 μM CCh for 100–120 min and monitored by time-lapse confocal microscopy. SEP-m2R staining allowed us to analyze and quantify the dynamics of membrane m2R disappearance only. GFP-m2R labeling experiments were useful to analyze the dynamics of m2R internalization (Figures 4A–G).

SEP-m2R fluorescence slowly drops to reach a minimum of 62% of initial levels 54 min after the application of the drug (Figures 3B,D). Then SEP-m2R labeling rises back to reach control values at 84 min after exposure to CCh. The statistical analysis shows a significant decrease of the m2R, 39, 54, and 69 min after the initiation of the treatment (Figure 3D). However, m2R fluorescence levels are not different from 39 to 69 min, revealing a steady state of the m2R intensity levels. Addition of NH4Cl reveals that m2R is associated with acidic compartments, by inducing the appearance of a strong punctiform labeling in the cytoplasm at the level of the soma and dendrites (Figures 3C,D).

While SEP-m2R fluorescence decreases at the PM, a punctiform GFP-m2R labeling appears close to the membrane and in the cytoplasm as early as 6 min after administration of CCh (Figure 4C). The statistical analysis demonstrates that the number of m2R clusters significantly increases as early as 10 min after the beginning of CCh treatment (Figure 4G). Time-lapse analysis shows that the number of internalized clusters increases with agonist exposure during the first 30 min and stabilizes afterward.

### The m2R is Associated With Structures Immunopositive for Native Clathrin After Stimulation With CCh

**FIGURE 5:**
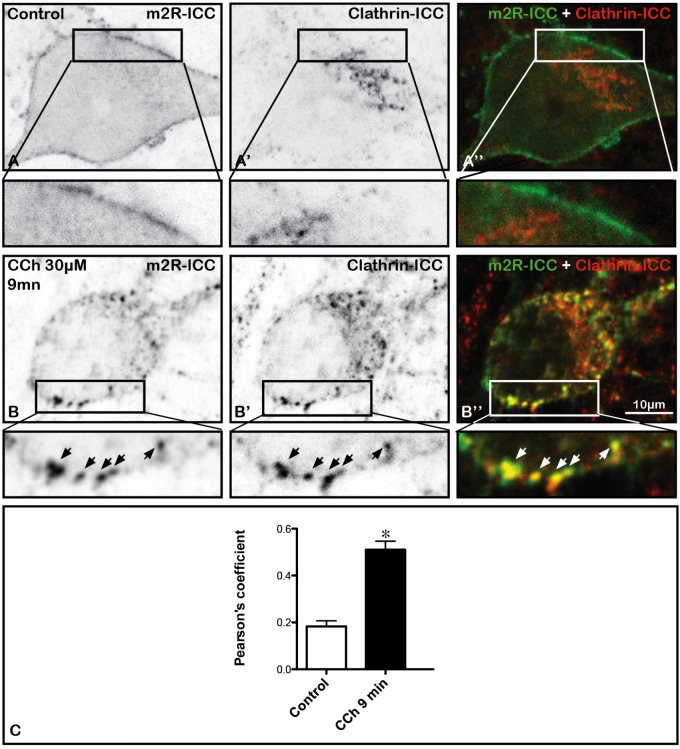
Internalization of m2R in native clathrin immunopositive structures in neurons after stimulation by CCh. Hippocampal neurons were transfected with a plasmid encoding the WT-m2R, stimulated with CCh (30 μM) for 9 min and fixed. A stack of 10 images were collected and an equatorial image was selected and an enlargement of a cell body is shown at the bottom of each image (insert). **(A–B”)** The m2R and Native clathrin immunopositive structures where co-detected by immunocytochemistry **(A’,B’)**. **(A–A”)** A control neuron displays no m2R and clathrin colocalization. Nine minutes after the initiation of CCh treatment, some m2R clusters colocalize with native clathrin immunopositive structures (arrows). **(C)** The analysis of the colocalization of clathrin and m2R-ICC was performed on fixed neurons using the Jacop Plugin of ImageJ are reported from the Costes’s randomization-based colocalization module (see Materials and Methods). The quantification of m2R and clathrin colocalization and the statistical analysis demonstrates a significant increase of the Pearson’s coefficient in treated neurons (*n* = 20) compared to cells treated with CCh (*n* = 20) (Mann–Whitney *U*-test; ^∗^*p* < 0.0001). Control neurons: *n* = 20; CCh-treated neurons: *n* = 21.

In order to determine whether the m2R may be found in intraneuronal structures containing native clathrin, we investigated the colocalization of m2R and clathrin immunofluorescence. In a control neurons, m2R rarely colocalized with clathrin (Figures 5A,A”). In contrast, in a neuron treated by CCh, m2R and Clathrin immunofluorescence often colocalized (Figures 5B,B”). The quantitative analyses of Pearson’s coefficients demontrated that colocalization of m2R and clathrin immunofluorescence significantly increased after CCh stimulation (Mann-Whitney *U*-test, ^∗^*p* < 0.0001; Figure 5C).

### Al594-Tf Uptake as a Marker of m2R Clathrin-Mediated Endocytosis

**FIGURE 6:**
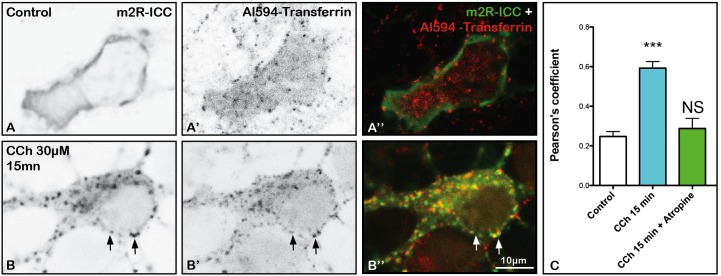
Al594-Transferrin (Al594-Tf) uptake and m2R clathrin-dependent endocytosis in hippocampal neurons. **(A–B”)** Hippocampal neurons were transfected with m2R-WT, pre-incubated with Tf-Al594, 10 min before CCh treatment. Cells were fixed after 15 min treatment. The m2R was detected by ICC. In control neurons, Al594-Tf is detected in the cytoplasm as a punctiform labeling **(A’–A”)**. CCh treatment induces a strong decrease of membrane m2R labeling and the appearance of m2R punctiform staining **(B,B”)**. Al594-Tf and m2R-ICC signal often colocalize (**B,B’, B”** arrows). The quantitative analysis of the colocalization of m2R and Al594-Tf in neurons was performed using the Jacop Plugin of ImageJ and statistical data are reported from the Costes’s randomization-based colocalization module (see Materials and Methods). Data are expressed as a Pearson’s coefficient (pc) and pc were compared using the Kruskal–Wallis test followed by the Dunn’s Multiple Comparison Test. Our analysis shows that the colocalization observed between the m2R immunofluorescent signal and Al594-Tf is higher after treatment with CCh compared to untreated neurons (^∗∗∗^*p* < 0.0001). Atropine prevents the increase of m2R and Al594-Tf colocalization (Atropine treatment vs. Control: NS, not significant). Control neurons: *n* = 25; CCh-treated neurons: *n* = 19; CCh-treated + atropine neurons: *n* = 9.

In order to know if m2R internalization involves CME, we analyzed uptake of a protein that is well known to display constitutive CME (Benmerah et al., 1999) in control neurons and in neurons treated with CCh. For that, the same neurons were incubated with Al594-Tf all along the experiment (see Materials and Methods). In a control neuron, Al594-Tf is detected in the cytoplasm (Figures 6A’,A”), whereas m2R is present at the PM (Figures 6A,A”). In contrast, in a neuron treated by CCh, m2R punctiform staining appear in the cytoplasm (Figures 6B,B” and m2R and Al594-Tf often colocalized (Figure 6B”). The quantitative analyses of Pearson’s coefficients, demontrated that colocalization of m2R immunofluorescence and Al594-Tf significantly increased after CCh stimulation (Kruskal–Wallis test, followed by Dunn’s Multiple Comparison Test: ^∗∗∗^*p* < 0.0001; Figure 6C). Atropine prevented the increased colocalization of m2R and Al594-Tf (Figure 6C).

### Blockade of m2R Clathrin-Mediated Endocytosis in Hippocampal Neurons

In order to determine if m2R internalization is strictly clathrin-dependent or whether it may involve other endocytotic pathways, we have used dominant-interfering mutant proteins (Eps15).

#### Expression of Eps15 Mutants Disrupts m2R Trafficking

**FIGURE 7:**
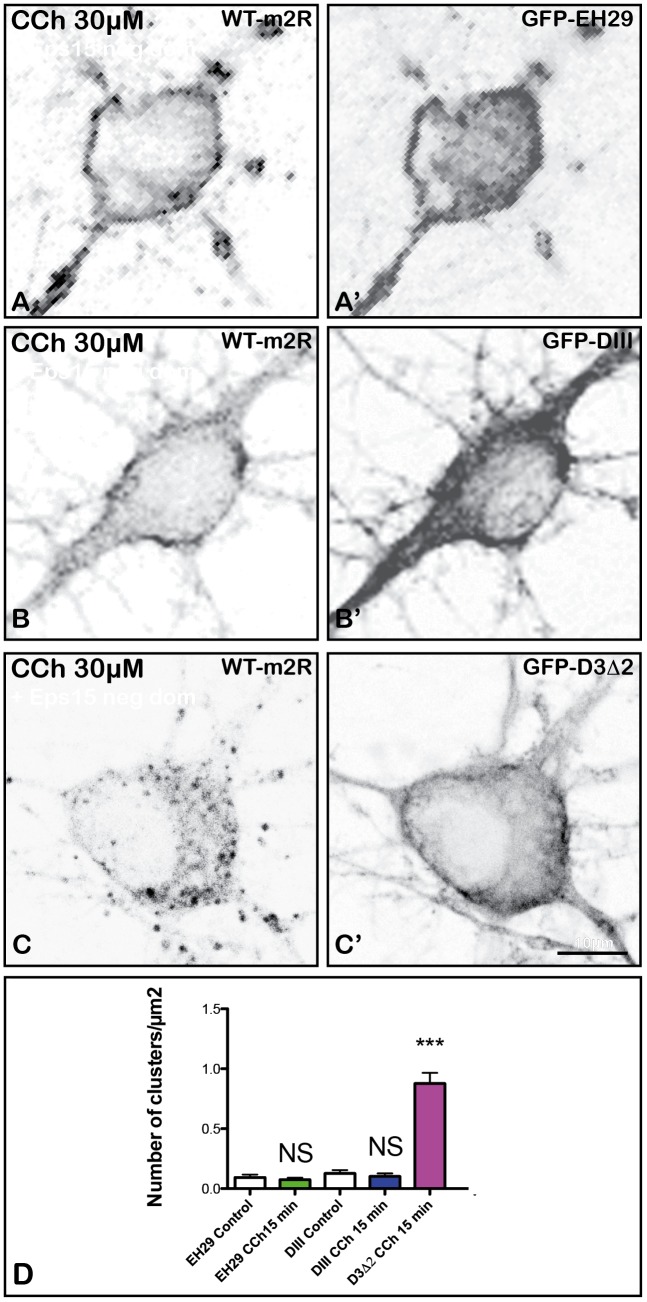
Blockade of m2R clathrin-dependent endocytosis in hippocampal neurons with negative dominant of Eps15. Hippocampal living neurons were co-transfected with WT- m2R plasmid and GFP-tagged EH29 and DIII mutants or their control (D3Δ2). The day after transfection, neurons were treated with CCh (30 μM) for 15 min and fixed. The m2R was detected by ICC. When the mutants are expressed (labeling in **A’,B’**), m2R labeling is seen at the plasma membrane of neurons **(A,B)**. The expression of the control plasmid (labeling in **C’**) does not block m2R internalization **(C)**. **(D)** Intracellular immunofluorescent clusters, representing m2R in endosomes, were segmented and counted using the FIJI/ImageJ software. Results are expressed as intracellular immunofluorescent clusters per μm^2^ cytoplasmic surface in Eps15 dominant negative-treated and control neurons. The statistical analysis shows that the expression of the EH29 and DIII mutants blocks m2R clusterization (Mann–Whitney *U*-test: NS, Not significant; EH29: Control neurons: *n* = 19; CCh-treated neurons: *n* = 19; DIII: Control neurons: *n* = 19; CCh-treated neurons: *n* = 30). In contrast, the control mutant (D3Δ2) does not inhibit m2R clusterization (Mann–Whitney *U*-test: ^∗∗∗^*p* < 0.0001; Control neurons: *n* = 19; CCh-treated neurons: *n* = 15).

Eps15 is a constitutive component of PM clathrin-coated pits (CCP) (Benmerah et al., 1999). To determine if disruption of Eps15 function influences m2R trafficking, we co-expressed in living hippocampal neurons two different Eps15 mutants (GFP-EH29 or GFP-DIII) or a control mutant, GFP-D3Δ2, with WT-m2R. We then analyzed m2R post-endocytic trafficking by confocal microscopy after a 15 min long CCh (30 μM) treatment. The expression of the mutants was checked by the detection of GFP staining in neurons (Figures 7A’,B’,C’). Expression of GFP-EH29 or GFP-DIII mutants completely prevents the punctate staining characteristic of m2R endocytosis induced by CCh. As shown in Figures 7A,B, m2R immunostaining is located mostly at the PM of soma and dendrites 15 min after the initiation of CCh treatment. In contrast, m2R is still internalized when the control mutant, GFP-D3Δ2, is expressed (Figure 7C). The statistical analysis (Figure 7D) confirmed that (1) GFP-EH29 or GFP-DIII mutants block m2R endocytosis (Mann–Whitney *U*-test: NS; Figure 7D) and (2) the control mutant has no effect on m2R internalization (Mann–Whitney *U*-test ^∗∗∗^*p* < 0.0001; Figure 7D).

### Role of Caveolin 1 in m2R Endocytosis

**FIGURE 8:**
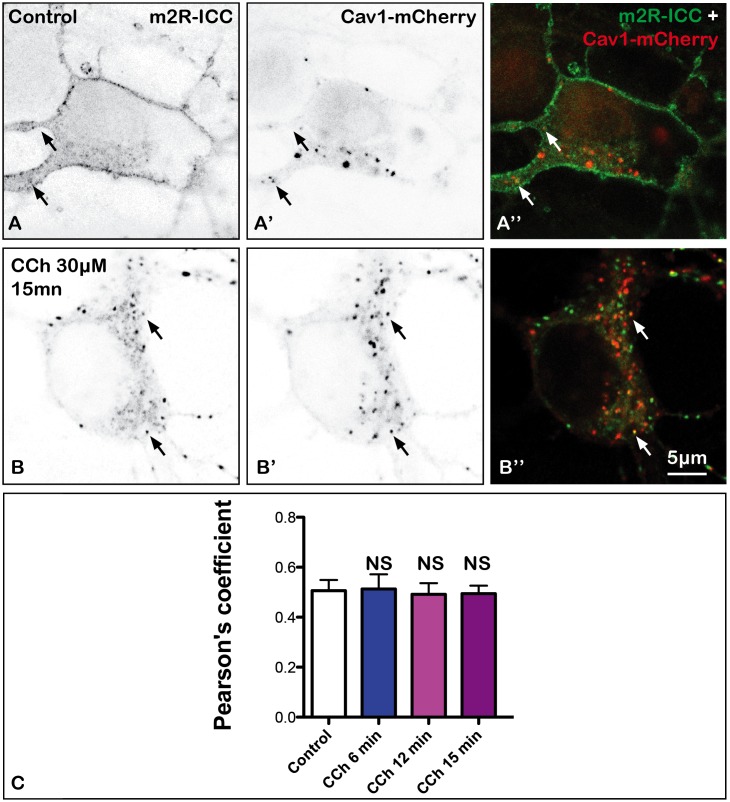
Absence of internalization of m2R in caveole in fixed neuron after stimulation by CCh. Hippocampal neurons were co-transfected with a plasmid encoding the wild-type receptor (WT-m2R: **A,B**) and CAV1-mCherry **(A’,B’)** fixed, and processed for visualization by confocal microscopy. In control and treated neurons, CAV1-mCherry is detected in the cytoplasm as a punctiform labeling **(A’,A”, B’,B”)**. Some m2R and CAV1-mCherry clusters colocalize (arrows) in both treated and untreated neurons. **(C)** The quantitative analysis of the colocalization of m2R and CAV1-mCherry in neurons was performed using the Jacop Plugin of ImageJ and statistical data are reported from the Costes’s randomization-based colocalization module (see Materials and Methods). Data are expressed as a Pearson’s coefficient (pc) and pc were compared using the Kruskal–Wallis test followed by the Dunn’s Multiple Comparison Test. Our analysis shows that pc values do not significantly differ in control neurons and neurons treated with CCh for 6, 12, and 15 min (NS, not significant).

Proteins may also internalize through clathrin-independent pathways (Doherty and McMahon, 2009). One of these pathways involves caveolae. To address the question of a role of caveolae-dependent pathway in m2R internalization, we have co-transfected the Cav1-mCherry plasmid with WT-m2R, and we have quantified colocalization of Cav1 and m2R fluorescent signals without or after 6, 12, or 15 min of treatment with CCh (Figure 8). We did not find any difference in Pearson’s coefficients between control and treated neurons (Mann–Whitney *U*-test: NS; Figure 8C).

### Post-endocytotic Fate of m2R

**FIGURE 9:**
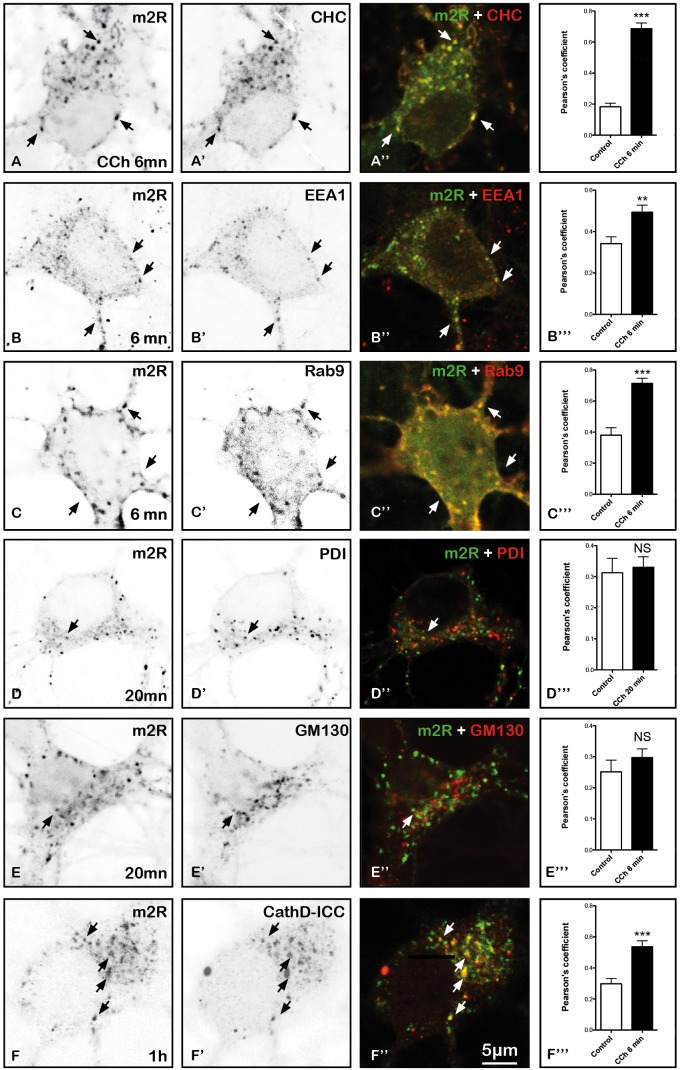
Immunohistochemical localization of m2R in neuronal compartments involved in endocytosis, synthesis, maturation, and degradation in fixed hippocampal neurons. Hippocampal neurons were transfected with m2R-WT. Neurons were stimulated with CCh at 30 μM for 6, 20 min and 1 h fixed, and processed for visualization of m2R together with markers of intraneuronal compartments and observed by confocal microscopy. (**A–C”)** 6 min after CCh stimulation (30 μM), some m2R immunopositive punta colocalize with CHC in clathrin-coated pits, EEA1 in early endosomes and Rab9 in late endosomes (arrow heads). **(D–E”)** Twenty minutes after CCh stimulation (30 μM), we failed to detect no colocalization of m2R with PDI, a marker of endoplasmic reticulum and GM130, a marker of Golgi apparatus. **(F–F”)** One hour after CCh stimulation (30 μM), some m2R immunopositive puncta colocalize with CathD, a marker of lysosomes (arrow heads). The quantitative analysis of the colocalization of m2R and markers of subcellular compartment in neurons was performed using the Jacop Plugin of ImageJ and statistical data are reported from the Costes’s randomization-based colocalization module (see Materials and Methods). Data are expressed as a Pearson’s coefficient (pc) and pc were compared using the Mann–Whitney *U*-test. Our analysis shows that the colocalization of the immunofluorescent signals for m2R with CHC, EEA1, Rab9, and CathD is higher after treatment with CCh compared to untreated neurons (CHC, Rab9, and CathD: ^∗∗∗^*p* < 0.0001; EEA1: ^∗∗^*p* < 0.01). In contrast, the colocalization of the immunofluorescent signals for m2R with PDI and GM130 do not significantly differ in CCh-treated neurons compared to untreated Control neurons: CHC *n* = 20, EEA1 *n* = 16, Rab9 *n* = 15, PDI *n* = 21, GM130 *n* = 17, CathD *n* = 17; CCh-treated neurons : CHC *n* = 12, EEA1 *n* = 18, Rab9 *n* = 15, PDI *n* = 20, GM130 *n* = 18, CathD *n* = 15.

Shortly after activation (6 min), m2R immunoreactivity is detected in numerous vesicles positive for CHC, a marker of clathrin immunopositive structures (CHC), EEA1 markers of early (EEA1) and late endosomes (Rab9) (Figures 9A–C”’) and cathepsin D, a marker of lysosomes (Figures 9F–F”). In normal neurons, as well as after 20 min of CCh exposure, m2R immunoreactivity does not overlap with PDI, a marker of ER or GM130, a marker of Golgi apparatus (Figures 9D–E”).

## Discussion

In the present study, we have developed a live-cell imaging approach to gain insights into the dynamics of a GPCR in living neurons, the muscarinic m2R. We have produced and validated different DNA constructs to allow expression of m2R in hippocampal neurons *in vitro*. We have especially studied the early steps of m2R endocytosis triggered by the stimulation. We have demonstrated, for the first time, that m2R is internalized in live neurons, after stimulation by an agonist, through a clathrin-mediated endocytotic pathway.

### Methodological Aspects

#### Structural Validation of Constructions

Consideration of receptor structure and function are important factors in the generation of fluorescent tag/GPCR chimeras. The construction has to preserve the native ability to address the m2R to the PM, to bind its ligands and not to modify intracellular receptor signaling. We have thus chosen to attach GFP at the extracellular N-terminus of the m2R, since this site is commonly used as a tagging site for many other GPCRs (McDonald et al., 2007b; Lelouvier et al., 2008). Several complementary experiments argue for the fact that our m2R-GFP and m2R-SEP constructs are valuable tools for such dynamic studies. Indeed, the m2R-GFP and m2R-SEP displayed the same subcellular localization as the wild type or the endogenous m2R (Bernard et al., 1998), i.e., is homogeneously distributed at the PM of the somatodendritic domain. With or without tag, most of m2Rs, revealed by GFP, or SEP or ICC, are detected at the PM, suggesting that the m2R is correctly addressed to the PM of soma and dendrites. Moreover, the easy detection of GFP and SEP at the PM using an anti-GFP antibody, in a non-permeabilized condition, demonstrates that the tag is correctly fused to the extracellular N-terminus of m2R protein. The colocalization of GFP or SEP fluorescence with anti-m2R ICC shows that GFP and SEP are faithful markers of m2R.

#### Validation of pH-Dependence o Time-Lapse Imaging Was Used to SEP-m2R

We used the SEP-m2R constructs to monitor and quantify variations of m2R at the PM upon agonist stimulation. Since SEP-m2R is tagged at the extracellular N-terminus, SEP will be present in the lumen of intracellular organelles during receptor endocytosis. Since these organelles have acidic pH (Demaurex, 2002), the fluorescence of endocytosed SEP-tagged receptors will be obscured. In agreement with these data, we have indeed demonstrated that (1) SEP-m2R fluorescence is quenched at acidic pH and (2) neutralization of intraneuronal vesicles medium by NH4Cl reveals their content in m2R. The SEP-m2R construct is therefore well suited to studying dynamic changes in surface receptor expression in live cells.

### Muscarinic Receptor Stimulation Induces m2R Internalization in Hippocampal Neurons

We have shown here that agonist stimulation of m2R induces internalization of this receptor in neurons *in vitro*. This is in agreement with previous data observed *in vivo* for the native receptor (Bernard et al., 1998; Liste et al., 2002; Decossas et al., 2003, 2005). Our results demonstrate that the incorporation of GFP into the m2R protein does not modify its ability to bind its ligands and internalize upon agonist stimulation. Indeed, m2R internalization induced by CCh stimulation was observed with the same timing (6 min after the initiation of activation) when GFP- and WT-m2R constructions are transfected. Moreover, we have checked that m2R internalization was actually due to specific activation of muscarinic receptors since it was blocked by atropine.

The study of receptor internalization phenomena requires the monitoring of two critical parameters: (1) the variation in receptor availability at the PM and (2) appearance of these receptors in intraneuronal compartments. The use of the SEP-m2R construct allowed the identification of three steps in the dynamics of membrane m2R density changes induced by agonist stimulation. First, m2R membrane density regularly decreases during the first 40 min. In the same time, GFP-m2R experiments demonstrates m2R clusters appearance in the somatic and dendritic cytoplasm. The use of NH4Cl on SEP-m2R expressing neurons reveals that the compartments containing m2R are acidic and thus probably correspond to endosomes. Taken together, our results suggest that this first step is mainly operated through endocytosis of membrane m2R into endosomes. Second, surface and internalized m2R densities stabilize (as measured by both SEP- and GFP-m2R). This suggests that the bulk internalization process is over. This may reveal a saturation of the endocytosis machinery, especially saturation of binding to protein involved in endocytosis. Lou et al. (2008) have shown that saturation of the endocytosis process occurs in absence of dynamin 1, a predominant component of the endocytic response. We may also assume that clathrin-dependent endocytosis has limited capacity and is saturated when m2R are saturated themselves as demonstrated for the EGF receptor (Schmidt-Glenewinkel et al., 2008). The third step is characterized by the recovery of m2R at the PM. At the same time, m2R is still accumulated in acidic compartments in the cytoplasm as demonstrated by NH4Cl application on SEP-m2R neurons and by the appearance of cytoplasmic GFP-m2R clusters. Recycling and/or neosynthesis of m2R may contribute to restore a normal receptor density at membranes. Recycling has been well studied for some GPCRs (Hanyaloglu and von Zastrow, 2008; Lelouvier et al., 2008; Zenko and Hislop, 2018) and was shown to be a key phenomenon in the recovery of cell function. Interference with each process through exposure to monensin (recycling) or cycloheximide (neosynthesis) should help to determine what is the mechanism leading to the normalization of surface m2R density.

### The m2R Endocytosis Is Clathrin-Dependent

We have brought here strong evidences for a clathrin-dependence of m2R endocytosis in neurons. First, we have shown that, shortly after stimulation, the m2R colocalized with clathrin immunopositive structures. Second, we have shown that m2R is partly internalized together with Al594-Tf, a molecule known to be internalized through a CME pathway. Third, disruption of CCP using over-expression of Eps15 negative dominants abolished m2R endocytosis.

The clathrin-dependence of m2R endocytosis is still under debate (Zenko and Hislop, 2018). Some studies demonstrated that m2R internalization pathway involves CCP only (Pals-Rylaarsdam et al., 1997; Jones et al., 2006; Yamanushi et al., 2007). Other claimed that m2R is internalized through a clathrin-independent process (Vogler et al., 1999; Delaney et al., 2002; Wan et al., 2015). Ockenga and Tikkanen (2015) propose that m2R endocytosis takes place by means of an atypical clathrin-mediated pathway that may involve a specific subset of CCP. Finally, some authors showed that the internalization of the m2R utilizes neither clathrin-coated pits nor caveolae (Roseberry and Hosey, 2001). These discrepancies may be explained in different ways. The signaling and trafficking properties of GPCRs may depend on the cell and cellular context (Ritter and Hall, 2009). Non-neuronal cells may not natively produce all the proteins involved in the clathrin mediated endocytotic machinery. In contrast, we have demonstrated that in neurons, m2R endocytosis is clathrin-dependent, even without overexpression of any endocytotic complex proteins. Indeed, we have shown agonist-induced m2R internalization in clathrin immunopositive structures.

Many studies have demonstrated the essential role of CCP in endocytosis and cellular signaling processes at the PM. CCP have also been shown to play a role in the transport of hydrolases from the Golgi complex to the lysosome and for polarity of the basolateral PM proteins in the epithelial cell line MDCK, and from the somato-dendritic membrane to axonal membrane in neurons (Deborde et al., 2008). We may hypothesize that clathrin immunopositive structures may play a role in the transport of endocytosed m2R from one subcellular compartment to another. Alternatively, clathrin was also shown to participate in rapid recycling after cargo accesses early endosomes (Zhao and Keen, 2008). Clathrin immunopositive structures containing m2Rs may thus contribute to recycling of m2R at the membrane, as we have suggested above.

### The m2R Does Not Involve Caveolae-Mediated Endocytosis

When m2R and CAV1-mCherry were co-expressed in the same neuron, we did not find that m2R clusters colocalized with CAV1-mCherry. This suggests that m2R endocytosis does not use the clathrin-independent pathway involving caveolae. Further experiments are required to determine whether other m2R undergoes other clathrin-independent endocytotic pathways.

### Post-endocytotic Fate of m2R

We have identified to which subcellular organelles m2R is targeted in order to identify the post-endocytotic pathway where the activated receptors are sorted (Figure 9). We have detected m2R in vesicles expressing Clathrin heavy chain (CHC), EEA1 and Rab9, as soon as 6 min after stimulation (Figures 9A–C”). This suggests that, after endocytosis in clathrin-coated pits (identified by CHC immunohistochemistry, Figures 9A–A”), m2R is sorted to early, then late endosomes (Figures 9B–C”). Early endosomes are considered as the first sites where internalized proteins, including GPCRs, are targeted before being either recycled, or degraded (Lakadamyali et al., 2003).

The colocalization of m2R with cathepsin D, a marker of lysosomes (Figures 9G–G”), confirms the hypothesis of m2R degradation after activation and endocytosis. This is in agreement with earlier data showing the accumulation of m2R into multivesicular bodies, which are organelles resulting of the fusion of lysosomes (Bernard et al., 1998; Tsuga et al., 1998).

The fact that m2R content is not increased in ER, revealed by PDI ICC in fixed neurons suggests that m2R is not targeted to compartments involved in m2R neosynthesis (Figures 9C–C”). This is in agreement with the absence of m2R-SEP labeling in live experiments. Indeed, if m2R-SEP is present in the ER, a neutral compartment, SEP should emit light. This is in agreement with earlier studies (Bernard et al., 1998).

## Conclusion

We have demonstrated here for the first time that m2R is endocytosed into living neurons via a clathrin-dependent pathway. The role of CME in signal transduction has yet to be fully understood. It is known that m2R, as an autoreceptor, modulates acetylcholine release in hippocampus and cortex (Zhang et al., 2002). How does m2R endocytosis alters acetylcholine release and is CME involved in this alteration are still open questions. It is likely that CME plays a key role in the regulation of signal transduction by physically removing activated m2R from the cell surface, that would have as a consequence to terminate the signal. Unless m2Rs recycle from endosomes. Another role of CME may be to produce transport vesicle to convey m2R to axonal varicosities where it is involved in the regulation of acetylcholine release. This phenomenon called transcytosis has been reported for Trk receptors (Ascano et al., 2009) but never for a muscarinic receptor. Analysis of m2R redistribution at the axonal levels may help to consider this hypothesis. If our work demonstrates that m2R is internalized through CME, we cannot exclude that another endocytotic pathway contributes to m2R internalization.

The regulation of m2R membrane availability may thus contribute to regulate neuronal sensitivity to acetylcholine and relative drugs in physiological or pathological conditions displaying abnormalities in acetylcholine transmission such as Alzheimer’s disease or schizophrenia (Wess et al., 2007).

## Author Contributions

LL has performed neuronal cultures, immunocytochemistry, and imaging. DD participated in immunocytochemistry experiments and analysis. AF participated to neuronal cultures. EH participated in molecular biology experiments. ZC, PD, and SEM contributed to data interpretations. VB conceptualized the research, designed the project, participated in the analysis, data interpretation, and drafted the work.

## Conflict of Interest Statement

The authors declare that the research was conducted in the absence of any commercial or financial relationships that could be construed as a potential conflict of interest.

## Abbreviations

ACh: acetylcholine
CCh: carbamylcholine
CCP: clathrin coated pits
GFP: green fluorescent protein
GPCR: G protein-coupled receptor
ICC: immunocytochemistry
LM: light microscopy
m2R: m2 receptor
NHS: normal horse serum
PBS: phosphate-buffered sodium
SEP: super ecliptic pHluorin
Tf: Alexa Fluor^®^ 594 Conjugated Tf.
